# The evolving strategies for the management of patients with metastatic gastric cancer: A narrative review and expert opinion

**DOI:** 10.3389/fmed.2022.1002435

**Published:** 2022-12-15

**Authors:** Stefano Cascinu, Maria Di Bartolomeo, Sara Lonardi, Giordano Beretta, Lorenzo Fornaro, Ferdinando De Vita

**Affiliations:** ^1^Comprehensive Cancer Center, IRCCS Ospedale San Raffaele, Università Vita-Salute San Raffaele, Milan, Italy; ^2^Department of Medical Oncology, Fondazione IRCCS Istituto Nazionale dei Tumori, Milan, Italy; ^3^Medical Oncology Unit 3, Veneto Institute of Oncology IOV - IRCCS, Padua, Italy; ^4^UOC Oncologia Medica, ASL Pescara P.O., Pescara, Italy; ^5^Unit of Medical Oncology, Azienda Ospedaliero Universitaria Pisana, Pisa, Italy; ^6^Oncologia Medica - Dipartimento di Medicina di Precisione, Università degli Studi della Campania Luigi Vanvitelli, Naples, Italy

**Keywords:** metastatic gastric cancer, immune checkpoint inhibitors, trifluridine/tipiracil, management, malnutrition, tumor markers

## Abstract

Gastric cancer (GC) is recognized as one of the most common deadly malignancies worldwide and about 40–50% of patients present at diagnosis with an unresectable disease due to a locally advanced or already metastatic condition. Recently, therapeutic options for management of metastatic GC (mGC) have been approved allowing a potential improvement of patient cancer treatment response and also an establishment of a continuum of care for this aggressive disease. This report is the result of a literature review by an expert panel. The aim of this document is to provide evidence, wherever it is lacking, to provide expert opinion directed at strategic management of mGC, and in particular aspect at practical management where appropriate guidelines are not available. Treatment landscape with new therapeutic strategies for third line and beyond, role of imaging, prognostic factors, symptoms, and markers as well as the importance of multidisciplinary approach particularly the nutritional aspects are discussed.

## Introduction

Gastric cancer (GC) remains a particularly aggressive disease. GC is the fifth most common malignancy and the third leading cause of cancer mortality worldwide, with no exception for Europe in which 136,038 cases and 96,997 deaths were estimated in 2020 (1, 2).

At diagnosis 40–50% of patients presents an unresectable disease due to locally advanced GC or metastatic spread (3). In presence of resectable disease, despite of the efficacy of peri-operative therapy and advances in surgical techniques, 40% of patients will relapse and long-term prognosis remains poor. Globally, the 5-year survival for GC is about 30% and has been stable for many years (4).

In western countries approximately 50% of patients presents with metastatic GC (mGC) at diagnosis. Of these, about 80% have a performance status score (PS) of 0–1 that allows the start of chemotherapy approach (4). In the last few years the landscape has been changing in the setting of mGC, albeit slowly. The implementation of biomarker testing, especially analysis of human epidermal growth factor receptor 2 (HER2) status, microsatellite instability status, and of the expression of programmed death-ligand 1 (PD-L1), had a significant impact on clinical practice and patient care. Moreover, it is estimated that about 40% of patients receiving first-line therapy may be candidate for second-line therapy, 15% of whom also for third-line one and beyond (5). There is evidence that patients who received multiple lines of therapy with nutritional support gained benefit in survival and quality of life (QoL) (6). Attention to these aspects has made it possible to increase the number of patients able to tolerate a pathway of sequential therapies and, consequently, able to obtain significant advantages in terms of symptoms control, survival, and QoL.

Due to mGC aggressive biological behavior, starting from the first treatment approach and during the implementation of therapies, a close patients monitoring is mandatory with the aim of identifying early and, hopefully, anticipating the possible clinical progression of disease (PD). In fact, an early overall patient disease assessment is necessary to avoid losing the most suitable chance for switching to subsequent lines of treatment. This results in a well-rounded evaluation, not limited to diagnostic imaging alone but also comprising the assessment of tumor markers, baseline prognostic factors as well as of symptoms and nutritional aspect, which must be considered equally relevant.

## Treatment landscape

### First line

Systemic chemotherapy represents the first therapeutic option for mGC with the aim not only of prolonging survival, but also of alleviating symptoms and improving QoL.

Regarding the standard chemotherapy schedules for the first-line approach, a Cochrane meta-analysis demonstrated, analyzing 4,447 patients randomized between mono-chemotherapy and multi-chemotherapy treatment from 23 studies, that combination regimens produce an advantage in term of overall survival (OS) compared to monotherapy (fluoropyrimidine alone) (7). In clinical practice, doublet combination with platinum-derived and fluoropyrimidine(s) [folinic acid, fluorouracil, and oxaliplatin (FOLFOX) or oxaliplatin and capecitabine (XELOX)] are recommended from international guidelines. One of the most debated issues is the potential role of a third drug in the combination chemotherapy. However, adding further drugs [e.g., docetaxel, cisplatin and 5-fluorouracil (DCF), modified DCF, folinic acid, fluorouracil, oxaliplatin, and docetaxel (FLOT)] is not recommended for metastatic disease. E.g., no survival benefit for FLOT over FOLFOX in patients over 65 years was documented, although a recent study reported that PS shows a stronger prognostic value than patient age in FLOT used as first-line therapy in a real-life cohort with advanced and mGC (8, 9). The role of a three-drug regimen over platinum-derived and fluoropyrimidine doublets was evaluated in a prospective randomized study which compared the combination of docetaxel, fluorouracil and cisplatin versus cisplatin and fluoropyrimidine. Although a triple advantage in terms of survival, response rate, and time to progression (TTP) was documented, the higher toxicity of the three-drugs regimen prevented its feasibility in most patients in daily clinical practice (10).

The folinic acid, fluorouracil, and irinotecan (FOLFIRI) scheme represents a valid therapeutic alternative. A French randomized phase III study compared the ECF triplet (epirubicin, cisplatin, and fluorouracil) to FOLFIRI, demonstrating a statistically significant time to treatment failure benefit and better tolerability of the combination with irinotecan (11).

For patients with HER2-positive disease, international guidelines for the treatment of unresectable locally advanced or mGC recommend the addition of trastuzumab to chemotherapy. Unfortunately, we have to consider that this advantage is limited only to the 10–15% of the patient population that overexpress this molecular target (12, 13). The role of biological anti-angiogenic drugs in the first line treatment for advanced GC has been evaluated in several randomized studies in both western and eastern populations. However, none of the two main studies demonstrated a significant benefit from adding anti-angiogenic agents to first-line chemotherapy (14, 15). A study that evaluates the role of combination of the ramucirumab and taxol as a switch strategy for maintaining response obtained with induction therapy with fluoropyrimidine(s) and oxaliplatin toward continuation of therapy with the same schedule is underway (16).

A new therapeutic scenario is emerging in the context of the first-line approach for GC with immunotherapy, alone or in combination with chemotherapy. The most relevant results come from the CheckMate 649 study which evaluated the addition of nivolumab to first-line chemotherapy. These results have led to the approval of nivolumab by both food and drug administration (FDA) and european medicines agency (EMA). Nivolumab is the first PD-1 inhibitor which showed superior OS, along with a progression-free survival (PFS) benefit and an acceptable safety profile, in combination with chemotherapy versus chemotherapy alone in treatment-naïve patients (17). Although FDA approval for nivolumab was granted regardless of PD-L1 status, the efficacy of the nivolumab-chemotherapy combination compared with standard chemotherapy regimens is evident in patients with high PD-L1 expressing tumor. Therefore, EMA recommended nivolumab for patients with tumor PD-L1 combined positive score (CPS) > or = 5. In the KEYNOTE-062 trial 763 patients were randomized to receive pembrolizumab alone versus chemotherapy plus pembrolizumab versus chemotherapy plus placebo. The results showed that immunotherapy alone is not inferior to chemotherapy alone, and the addition of pembrolizumab to chemotherapy does not translate into significant advantage in terms of survival (18).

Immunotherapy seems to play a role even in HER2-positive tumors. In fact, the combination of pembrolizumab with trastuzumab and chemotherapy showed a significant improvement in response rate in the KEYNOTE-811 trial (19).

### Second line

The role of second-line therapy in mGC has been under debate among oncologists for several years due to the risk of exposing patients with suboptimal PS (for high tumor burden) to potentially toxic agents. As a result, no more than 10 years ago, only 14% of patients enrolled in western clinical trials compared to 75% of patients enrolled in Asian trials received a second-line treatment (20, 21). Only in more recent years the definitive demonstration that a rescue therapy is able to improve the survival and QoL of these patients was obtained. This changed the oncologists’ paradigm of treatment, representing the prerequisite for thinking about a continuum of care strategy also for mGC. The evidence supporting the use of salvage chemotherapy for patients who progress after first-line treatment has been based on some phase III trials which have demonstrated the feasibility of a second-line treatment. In particular, in these series, both irinotecan and taxanes (docetaxel and paclitaxel) alone were associated with a significantly longer survival compared to placebo without a significant difference between the agents used in the experimental arms (22–24). The data were confirmed by a subsequent meta-analysis which showed the benefit of a second-line treatment with a hazard ratio (HR) of 0.75. In particular, the analysis demonstrated an increase in OS in favor of the arm with an additional cytostatic or targeted therapeutic agent (25).

However, thanks to the results of the REGARD and RAINBOW studies, second-line therapy became a standard approach able to entail a continuum of care similarly to the one already achieved for other malignancies. The REGARD study compared ramucirumab, a monoclonal antibody directed against VEGFR2, versus best supportive care (BSC) in 355 patients progressing after the first-line approach. A significant advantage was observed in terms of OS (primary objective) with a gain of 1.4 months (HR 0.77), but also in terms of PFS and disease control rate (DCR), with a particularly acceptable toxicity profile (26). In the RAINBOW study, conducted in the same setting represented by 665 patients, the combination of ramucirumab and paclitaxel was compared to paclitaxel alone and showed a statistically significant benefit in terms of OS, PFS, and DCR; in particular, there was a gain of 2.3 months in OS (HR 0.80) in the experimental arm, which was statistically significant and clinically relevant (27). Furthermore, in both studies, the investigational treatment also appeared to be able to impact on the QoL of patients, producing a significant delay in the time to deterioration of the PS (28). The results of these two studies, which unquestionably represent the most consistent evidence in supporting a second-line approach in mGC, have certainly positively influenced clinical practice and definitively established the role of second line therapy, demonstrating how ramucirumab alone or in association with paclitaxel is able to prolong survival and improve QoL by providing adequate palliation of symptoms. However, the particular frailty of GC patients requires careful monitoring by physicians and an early nutritional assessment to optimize the therapeutic strategy, expanding the number of patients who are really able to benefit from a sequential therapeutic approach.

Recently, in the long-term follow-up of the phase II multicohort KEYNOTE-158, pembrolizumab confirmed durable antitumor activity in 233 patients with previously treated advanced microsatellite instability high (MSI-H) or mismatch repair deficient (dMMR) advanced solid tumors (29). In fact, EMA approved pembrolizumab for patients with instability and with treatment failure on or intolerance to standard first-line therapies (30).

### Third line and beyond

The improvements that have been obtained in recent years in the clinical management and treatment of patients with GC have led to an increase in the number of patients who, after progression to the second-line approach, still maintain fair general conditions and are therefore potentially able to receive further lines of therapy. It is estimated that these patients can represent about 20% of patients with mGC (31, 32). In these patients, the availability of treatments which can contribute with a benefit in terms of TTP, maintenance of general conditions and increased survival compared to supportive therapy alone may be particularly relevant. On the other hand, in patients who present poor general clinical conditions, the availability of further treatments is superfluous since, in these cases, the correct indication remains best supportive therapy.

Benefits were observed with docetaxel or irinotecan, but no scientific evidence supports such use and data on risk/benefit and cost/benefit are still lacking. The use of these drugs is in fact associated with numerous toxicities including nausea, vomiting, asthenia, diarrhea and abdominal pain, which potentially compromise QoL (33, 34).

A phase III trial and a real-world analysis conducted in China demonstrated a benefit with apatinib, a novel, orally administered VEGFR inhibitor, in the third-line setting (35). Nevertheless, a randomized phase III trial including both Asian and western patients failed to confirm this benefit in patients with advanced/mGC who failed at least two prior chemotherapy regimens (36). To date, apatinib is not still approved by FDA and EMA.

In third or later line treatment, two checkpoint inhibitors were evaluated. In a phase III study (ATTRACTION-2) conducted in Japan, Republic of Korea, and Taiwan, median OS was 5.3 months in the nivolumab group versus 4.1 months in the placebo group (HR 0.63, *P* < 0.0001) (37, 38). In a large phase II study (KEYNOTE-059), median OS was 5.8 months in patients with PD-L1-positive disease, who received pembrolizumab (39). Nevertheless, these checkpoint inhibitors did not receive EMA approval, apart from pembrolizumab in patients with MSI-H or dMMR with treatment failure on or intolerance to standard first-line therapies (30).

The only drug that more recently has been shown to be statistically and clinically significant at improving third-line survival in patients with mGC is trifluridine/tipiracil, an oral anti-neoplastic agent consisting of trifluridine, a thymidine-based nucleoside analog, and tipiracil, a thymidine phosphorylase inhibitor, which improves the bioavailability of trifluridine (40). Based on the results of the phase III TAGS study, in fact, the european society for medical oncology (ESMO) guidelines were specifically updated in November 2019 after the approval of trifluridine/tipiracil by EMA. ESMO guidelines recommend trifluridine/tipiracil therapy as the only standard in this setting with a IA level of evidence (41–43). Regulatory approvals were due to an advantage in terms of both PFS (2.0 versus 1.8 months, HR 0.57) and OS (5.3 versus 3.6 months, HR 0.69), showing for the first time that offering a third line to patients with GC and good general conditions can lead to an overall improvement for patients. Trifluridine/tipiracil demonstrated a fully manageable toxicity profile in heavily pretreated mGC patients: adverse events were generally easily managed by dose modifications and/or supportive care. Another aspect in favor of trifluridine/tipiracil is the evidence of the statistically and clinically significant effect on time to PS deterioration: it seems to be possible to deduce that many patients maintain a PS score of 0–1 even after progression, thus becoming potentially candidates for other, hopefully, treatment lines that will be available in the future. These data acquire even more value considering that patients are also accompanied by a maintenance of QoL.

The survival benefit of trifluridine/tipiracil in the TAGS study is similar to the one showed by pembrolizumab and nivolumab in the same setting but, to date, trifluridine/tipiracil is the only agent approved by both FDA and EMA.

In addition, an exploratory analysis of the TAGS study showed efficacy of trifluridine/tipiracil in both third and later lines in terms of OS, PFS and in improving median time to deterioration to a PS score ≥ 2 compared to placebo. Further analyses reported a numerically higher efficacy of trifluridine/tipiracil in third line compared to the fourth one. The survival benefit in third line materializes in a median OS of 6.8 months (6.0 months for patients with peritoneal metastases), a median PFS of 3.1 months and in a median time to deterioration to a PS score ≥ 2 of 2.8 months (44). Taking in consideration that the fourth line is difficult to reach by mGC patients, these results confirm the key essential role of trifluridine/tipiracil in third-line setting and further support the updated guidelines for the use of the drug in patients with mGC (13).

## Disease assessment

With the improvement of imaging technology, most GCs can be basically diagnosed through electronic gastroscopy, gastrointestinal angiography, gastroscopy ultrasound, computed tomography (CT), magnetic resonance imaging, and positron emission tomography (45). To assess and promptly identify signs and/or symptoms of PD, oncologists have to scheduled routinely CT scan assessment with an appropriate timing and add extra-assessment in case of suspected PD. Timing for appropriate CT scan should be based on patient therapeutic journey. In details, as PFS tends to decrease with the number of therapies, a detailed disease re-evaluation has to be performed more frequently in a patient who is undergoing third-line approach respect to a patient who is in the first-line one.

When there is a suspicion of PD, even if signs of disease progression are not detected through the most recent CT evaluation, clinicians should consider the use of another radiographic assessment modality such as barium enema, or ultrasonography, to determine whether peritoneal metastasis is present (45). However, sometimes a peritoneal progression might be not radiologically evident even using more than one technique, therefore physician should be aware to carefully evaluate symptoms worsening and carcinoembryonic antigen (CEA) or CA19-9 increase to consider a clinical progression and to decide for a subsequent line of treatment. Peritoneal metastasis, which is the most common form of recurrence in GC, is estimated to occur in 55–60% of GC patients thus this issue is of particular importance.

In some cases, conventional guidelines for response assessment are not suitable for the evaluation of response to immunotherapy. To note, excessive adherence to RECIST criteria may result in missing the appropriate timing for switching to second- or third-line therapy (46). Recently, new criteria, iRECIST, have been proposed specifically for immunotherapy (47).

To date, though RECIST criteria are important in terms of response evaluation, clinical decisions should not be based exclusively on radiologic findings but should take into consideration other issues.

## Tumor markers

In clinical practice tumor markers are useful tools in some situations to monitoring disease. Their levels in the blood can be used for the tracking of how effectively cancer treatments are working or if cancer has come back.

To date, an increase in tumor markers levels should not be the unique indication that implies therapy interruption or switching to another agent. The markers oncologist mostly refer to are CEA and CA19-9 (48, 49), but often a small change in diagnostic imaging can be found when the level of tumor markers increases in mGC (46). In particular, the CEA-positive patients had larger tumors, more frequent lymphatic and vascular involvement, and higher rates of lymph node and hepatic metastases (49).

Circulating tumor DNA (ctDNA) is a tumor-derived fragmented DNA in the bloodstream that has come from primary or metastatic cancer sites. Liquid biopsy and other new ctDNA technologies represent a paradigm shift in cancer diagnostics because they can be used to monitor the tumor response to neoadjuvant and postoperative therapy in patients with mGC. Using clearance of ctDNA as an endpoint for escalation/de-escalation of adjuvant chemotherapy in patients considered to have high-risk disease has become an important area of research and it may be of help also in the advanced setting in order to choose the best time to switch to another therapeutic regimen (50). This could indicate a new useful tool also for the other mGC therapies as ctDNA can detect disease recurrence several months prior to imaging with a potential impact on survival.

MSI-H or dMMR are strongly predictive of immunotherapy benefit, regardless of number of therapies already received. MSI-H/dMMR is detected in up to 8% of patients with mGC. Early treatment with checkpoint inhibitors may be particularly beneficial in this patient population but data are limited by the relative rarity of MSI-H/dMMR disease (30, 51).

Covalently closed circular RNAs (circRNAs) have emerged as crucial regulators in several human cancers including GC. To date, various circRNA candidates have been validated and engaged as GC metastases markers. However, a global and comprehensive understanding of circRNAs related to GC metastases is still scarce. To gain better and deeper insight into the aberrant expression pattern of circRNAs involved in GC metastases, genome-wide circRNA profiling with high throughput sequencing from mGC tissue could be a powerful approach to address this issue (52).

MiRNAs are altered in GC, showing activity as both tumor suppressors and oncogenes, although their true roles have not been fully understood. MiRNAs are associated with GC development and progression, tumor microenvironment and chemoresistance and further research is needed examining for assess the specific impact on GC (53).

Recently, the modified japan clinical oncology group (JCOG) prognostic index (which incorporated diffuse-type histology and high neutrophil-to-lymphocyte ratio level into the JCOG prognostic index) showed excellent stratification of OS in real-life patients, as it could also help determine the need for treatment changes throughout the patient cancer journey (54). Other prognostic factor analyses/nomograms can be used to aid clinical decision-making in first-, second-, and third-line settings (Table 1) (32, 55–57).

**TABLE 1:** Summary of currently available treatment options and major clinical prognostic determinants in metastatic gastric cancer.

Treatment line	Major negative prognostic factors	Molecular determinants	Chemotherapy regimens	Targeted agent or immune checkpoint inhibitor	References
First	ECOG PS≥ 1 ≥ 2 metastatic sites no prior gastrectomy abnormal serum ALP peritoneal metastases liver metastases	Mandatory: HER2 (IHC, FISH) PD-L1 CPS (IHC) Recommended: MMR/MSI (IHC, PCR)	Preferred: Platinum + fluoropyrimidine Alternative: FOLFIRI DCF, modified DCF, FLOT	Available: Trastuzumab (HER2-positive disease; in combination with cisplatin and 5-fluorouracil or capecitabine) Nivolumab (PD-L1 CPS ≥ 5, HER2-negative disease; in combination with platinum and fluoropyrimidine)	(7, 9–11, 13, 17, 20, 54, 55, 58, 59)
Second	ECOG PS ≥ 1 peritoneal metastases neutrophil-lymphocyte ratio abnormal serum LDH first-line PFS < 6.8 months	Recommended: MMR/MSI (IHC, PCR) (if not available)	Docetaxel Paclitaxel Irinotecan	Available: Ramucirumab (in combination with paclitaxel, after progression on platinum and fluoropyrimidine in first-line; as monotherapy, after progression on platinum or fluoropyrimidine in first-line) EMA-approved: Pembrolizumab (MSI-H or dMMR disease, progressing on or following at least one prior therapy)	(22–24, 26, 27, 29, 56–58)
Third	ECOG PS ≥ 1 first-line PFS < 6.9 months second-line PFS < 3.5 months	Recommended: MMR/MSI (IHC, PCR) (if not available)	Preferred: Trifluridine/tipiracil Alternative: Docetaxel Paclitaxel Irinotecan (if not received previously)	EMA-approved: Pembrolizumab (MSI-H or dMMR disease, progressing on or following at least one prior therapy)	(29, 31–34, 41)
Beyond third	ECOG PS ≥ 1 first-line PFS < 6.9 months second-line PFS < 3.5 months	Recommended: MMR/MSI (IHC, PCR) (if not available yet)	Preferred: Trifluridine/tipiracil Alternative: Docetaxel Paclitaxel Irinotecan (if not received previously)	EMA-approved: Pembrolizumab (MSI-H or dMMR)	(29, 31, 32, 41)

ALP, alkaline phosphatase; DCF, docetaxel, cisplatin and 5-fluorouracil; dMMR, mismatch repair deficient; ECOG PS, eastern cooperative oncology group performance status; FLOT, folinic acid, fluorouracil, oxaliplatin, and docetaxel; IHC, immunohistochemistry; LDH, lactate dehydrogenase; MMR, mismatch repair; MSI, microsatellite instability; MSI-H, microsatellite instability high; PFS, progression-free survival; PCR, polymerase chain reaction; FISH, fluorescence in situ hybridization.

Thus, monitoring of markers is of importance in the overall workup of mGC.

## Symptoms

Symptoms may play a crucial role in the management of mGC patients. If clinical symptoms or abnormal blood tests (e.g., renal dysfunction, elevated bilirubin or elevated tumor markers levels, alkaline phosphatase or lactate dehydrogenase) suggest for an exacerbation of the disease, imaging has to be carried out. Results can determine a shift to a further treatment after a recommended discrimination between treatment toxicities and disease progression.

The most common presenting symptoms for GC are non-specific weight loss, persistent abdominal pain, dysphagia, hematemesis, anorexia, nausea, early satiety, and dyspepsia. Patients presenting with a mGC usually present with significant abdominal pain, potential ascites, weight loss, fatigue, and have visceral metastases on scans, and can have a gastric-outlet obstruction.

## Nutrition

Screening and assessment of malnutrition at diagnosis, its monitoring during the therapeutic pathway, early detection of pre-cachexia and the ongoing use and consultation of a multidisciplinary team are effective weapons for oncologists (6). A patient who reports weight loss at the start of chemotherapy is destined to show a worse survival. On the contrary, identifying a condition of malnutrition early and correcting it promptly, results in an improvement in the patient prognosis; it is evident that all this implies the possibility for the patient to maintain a better PS and QoL and therefore to be able to benefit from more lines of treatment in a sequential manner. Patients with mGC often suffer from malnutrition, which can have an impact on QoL, increase the toxicity of chemotherapy and reduce OS (58). Malnutrition is often overlooked and undertreated in clinical practice despite being very common in GC and having a negative impact on patients. In 2016, the global leadership initiative on malnutrition proposed a two-step approach for diagnosing malnutrition that entailed: screening to identify risk status, assessing for diagnosis and grading the severity of malnutrition (weight loss and muscle loss resulted as key phenotypic criteria for malnutrition) (59). Another study evaluated mGC patients who had started first line chemotherapy: 105 out of 118 patients (89%) had baseline sarcopenia and 31% developed muscle loss during chemotherapy. Results showed that muscle loss was significantly associated with shorter time to failure and OS, and that it was an independent prognostic factor for both these parameters (60).

Despite the recent improvement in the treatment of GC, including antiangiogenic therapies and immunotherapy, nutritional care for GC lags substantially compared to what happens for other cancer types. Furthermore, while many studies have investigated perioperative nutritional care for patients with GC who have undergone gastrectomy (61), there is limited literature regarding nutritional care for patients with advanced or mGC and therefore general guidance should be followed (62). In fact, available data, research and guidelines addressing this issue vary considerably, and many malnourished patients do not receive adequate nutritional care. Studies have revealed that poor nutritional status is an important negative prognostic factor for patients with mGC and malnutrition, cachexia and sarcopenia, to some degree, all have a negative impact on the QoL of patients with GC (6, 58, 63). Ultimately, cachexia is responsible for over 20% of all cancer-related deaths (64).

In addition, the dose of anticancer drugs is usually calculated based on patients’ body surface area or body weight without regard to any changes in body composition (e.g., proportions of muscle, fat, and water) (65, 66). A critic issue is that malnutrition can impact patients’ body composition resulting in an excess of toxicity from anticancer drugs and, consequently, leading to a reduced dose of therapy or delayed treatment cycles with loss of efficacy.

All patients should be screened for malnutrition at the time of initial diagnosis of mGC, and it should be determined whether there is a nutritional risk, such as weight loss, anorexia, sarcopenia or cachexia, low body mass index and/or systemic inflammation. If the patient is found to be at risk, a full nutritional assessment should be conducted by a dietitian/nutritionist (67, 68).

For patients without malnutrition at baseline, this approach will help in the early identification of patients at risk of malnutrition and allow prompt treatment and/or a careful follow-up. In fact, during the course of anticancer therapy, patients’ nutritional status often changes due to the worsening of underlying disease and associated symptoms and/or toxicities induced by anticancer therapies. Therefore, evaluation of nutritional status should be performed on a regular basis and at short time intervals (2–3 weeks) throughout the whole cancer journey ensuring timely clinical interventions.

To provide improved clinical outcomes for patients with mGC, a multidisciplinary team (e.g., gastroenterologists, dieticians/nutritionists, surgeons, pain specialists, nurses, and psychologists) to allow provision of the most appropriate nutritional care is recommended (67).

## Discussion and expert opinion

Metastatic GC represents a biologically aggressive disease: therefore, during the implementation of treatments, starting from the first line, close monitoring of the patient is necessary with the aim of identifying early and to anticipate the possible clinical PD. The aim is to avoid loss of the most suitable time window for switching to further lines of therapy. This means that the patient’s follow-up during the treatment cannot be entrusted exclusively to diagnostic imaging, but that clinicians must also consider bio-humoral tests (in particular tumor markers), the baseline prognostic factors and, in particular, the symptoms manifested by the patient. Equally relevant is the evaluation of the nutritional aspect: a patient who exhibits weight loss during the start of chemotherapy is a patient destined to show a worse survival. On the contrary, identifying a condition of malnutrition early and correcting it promptly result in an improvement in the patient’s prognosis; it is evident that all this implies the possibility for the patient to maintain a better PS and therefore to be able to benefit from more lines of treatment in a sequential manner.

All these aspects take on a very significant value because they represent one of the most important contributions to the improvement of treatment outcomes for mGC patients, in parallel with the evolution of available drugs. Palliative management, which may include systemic therapy, chemoradiation, and/or BSC, is recommended for all patients with unresectable or mGC.

However, it is important to emphasize that all improvements have led, also in light of the approval for the third line and beyond of trifluridine/tipiracil, to be able to extend the concept of continuum of care to mGC patients and thus to increase survival. The greater survival in the third-line setting obtained with trifluridine/tipiracil lends further validity to guideline recommendations and supports efficacy in a broader patient population.

**FIGURE 1:**
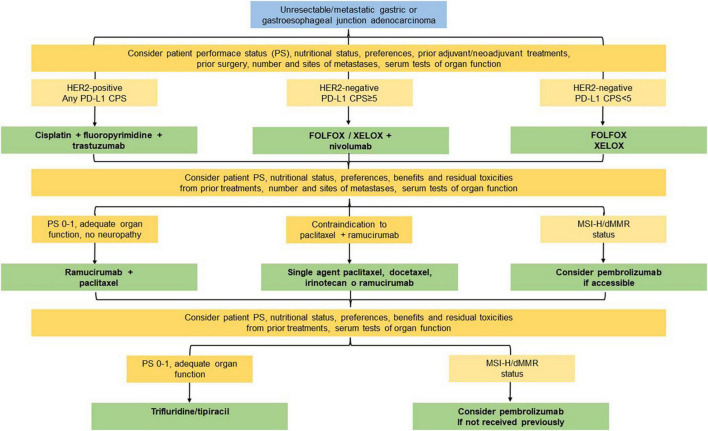
Expert recommendations for the optimal management of metastatic gastric cancer.

To sum up, we propose an overview for the optimal management of mGC taking into account all the aspects aforementioned which can be resumed in Table 1 and Figure 1, resulting in practical recommendations for deciding treatment protocol.

In conclusion, although the treatment of GC slowly produces results, the last decade has unquestionably seen an improvement in the management and therapeutic strategies in both operable and advanced disease. In particular, in mGC the chance of implementing a continuum of care strategy by a sequentially pattern of different active treatments has made it possible to improve the prognosis of patients. The optimization of supportive therapies, especially nutritional ones, contributed to this goal: early identification of malnourished patients or patients at risk of malnutrition allows to improve the control of symptoms, QoL and PS of patients, to offer them subsequent lines of treatment.

Metastatic GC today represents one of the most important unmet medical needs in Oncology. The awareness of having to consider management from various points of view and the new approved therapies will lead to an overall significant improvement in the care strategy of this critical patient setting.

Recent advances in the clinical management of the disease have led to an increase in the number of patients who, after progression to the second line, maintain good general conditions (PS and QoL) and can therefore benefit from a third line of therapy (and beyond?).

## Author contributions

SC and FD wrote the manuscript. MD and SL collected the data. GB and LF analyzed the data. All authors contributed to the article and approved the submitted version.
